# Evaluation of Symmetry and Morphology in Mandibular Permanent Anterior Teeth Using Cone‐Beam Computed Tomography in an Iranian Population

**DOI:** 10.1155/ijod/9659582

**Published:** 2026-08-20

**Authors:** Narges Simdar, Javad Mesri, Seyed Mohsen Helmi

**Affiliations:** ^1^ Department of Endodontics, Faculty of Dentistry, Guilan University of Medical Sciences, Rasht, Iran, gums.ac.ir; ^2^ Department of Cosmetic and Restorative Dentistry, Faculty of Dentistry, Mashhad University of Medical Sciences, Mashhad, Iran, mums.ac.ir

**Keywords:** canine, central incisor, cone-beam computed tomography, lateral incisor, mandibular anterior teeth, morphology, symmetry

## Abstract

**Introduction:**

Achieving successful root canal treatment requires detailed understanding of the root canal. A clear and detailed understanding of the root canal anatomy of mandibular anterior teeth is of great importance for endodontists. Several techniques have been introduced for evaluating root canal morphology, including cone‐beam computed tomography (CBCT). The current study applied CBCT to evaluate the root canal configurations and symmetry patterns in the lower anterior teeth in an Iranian sample.

**Materials and Methods:**

In this descriptive cross‐sectional study, 148 CBCT images from 148 patients at the Oral and Maxillofacial Radiology Clinic were chosen on the basis of predefined inclusion and exclusion criteria. A total of 852 permanent mandibular anterior teeth were examined to determine the number of roots and canals, and the canal morphology was categorized on the basis of Vertucci’s system, as well as symmetry, utilizing CBCT image analysis. The results were compared based on gender and the right versus left side of the mandible.

**Results:**

Participant ages ranged between 16 and 60 years, with an average age of 36.76 ± 9.67 years; 44.6% were male and 55.4% were female. Both the lateral and central incisors consistently showed a single root, whereas 3.2% of canines had a secondary root. Second canals were found in 32.6% of centrals, 29.7% of laterals, and 6.1% of canines. The most commonly observed type of Vertucci root canal morphology was type I, followed by type III for incisors, while type I was also predominant in canines. The symmetry rates for centrals, laterals, and canines were 90.1%, 93.1%, and 91.4%, respectively. There were no significant differences in the number of roots and canals, morphological frequency, or symmetry between female and male patients or between both sides of the mandible.

**Conclusion:**

Mandibular anterior teeth predominantly exhibit single‐rooted structures with one root canal. Vertucci type I morphology is the most prevalent among these teeth, with morphological frequency unaffected by jaw side or sex. Additionally, the findings indicate a high degree of symmetry among mandibular anterior teeth, with no significant differences between genders.

## 1. Introduction

Recent advancements in cone‐beam computed tomography (CBCT) technology have significantly improved the accuracy of root canal system visualization, enabling clinicians to detect complex anatomical variations with greater precision [1]. The fundamental purpose of endodontic treatment is thorough disinfection and debridement of the entire canal system [2]. Achieving this goal necessitates a comprehensive understanding of root canal morphology [3]. Recent studies emphasize that the morphology of the canals shows notable variations across different ethnic populations [4]. One of the most common factors contributing to unsuccessful root canal treatment is the inability to effectively clean the entire root canal system [5]. Studies indicate that ~60% of endodontic failures are attributed to insufficient cleaning, while 20% result from a failure to recognize multiple canals [2, 3, 5].

Lower anterior teeth are typically single‐rooted and single‐canaled [6]. Lower centrals and laterals show very similar canal anatomy; however, the spatial configurations of the canals may vary based on factors such as ethnicity, race, and gender [6–8]. A recent meta‐analysis by Martins et al. [9] highlighted that mandibular incisors tend to have a higher frequency of second canals in Asian populations compared to Caucasian populations, underscoring the importance of population‐specific studies. Contemporary research using CBCT has demonstrated superior accuracy in detecting fine anatomical variations [3, 10]. Miyashita et al. [7] and Ezoddini et al. [8] indicated that 15% and 55.9% of the lower incisors, respectively, exhibited a second root canal. Vertucci [11], Sert and Bayirli [12], and Al‐Qudah and Awawdeh [13] implied that 27.5%, 68%, and 26.2% of the lower incisors had a second root canal.

CBCT provides accurate assessments of the structure of bone and teeth from various angles [14]. The advantages of CBCT include low radiation exposure for patients, short examination times, and high‐resolution imaging [14, 15]. According to Scarfe et al. [16], CBCT is now considered the gold standard for preoperative assessment of root canal anatomy due to its ability to provide three‐dimensional reconstructions with minimal distortion. These features have contributed to the increasing utilization of CBCT in dental practice [17].

To date, no comprehensive study has evaluated the root symmetry and morphology in anterior teeth within the Iranian population. Therefore, this study was performed to assess the root symmetry and morphology of lower anterior teeth by means of CBCT.

## 2. Materials and Methods

This retrospective descriptive cross‐sectional study was conducted on CBCT images of patients attending the Oral and Maxillofacial Radiology Department of Guilan University of Medical Sciences between 2018 and 2021. The prescription for CBCT imaging was part of the patients’ treatment processes, including implant placement, surgical procedures, and orthodontics. The research received ethical approval from the Ethics Committee of Guilan University of Medical Sciences (IR.GUMS.REC.1399.358). Owing to the retrospective nature and the use of anonymized preexisting images, the need for informed consent was waived.

A retrospective convenience sampling method was used to select 148 CBCT scans that fulfilled the following inclusion criteria: presence of mandibular anterior teeth with complete root development, no previous endodontic treatment, absence of periapical lesions, no internal or external root resorption, no extensive or deep caries, and no calcified canals. This approach was chosen due to the availability of preexisting images taken as part of routine clinical care. Altogether, 852 mandibular anterior teeth were evaluated, comprising 280 canines, 290 lateral incisors, and 282 central incisors from 148 patients. There were no missing data for any of the study variables, as all included CBCT images met the predefined inclusion criteria and contained complete information regarding root and canal morphology. The selection process of the CBCT scans is illustrated in Figure S1.

All scans were acquired using a NewTom Giano CBCT unit (QR srl, Verona, Italy) with the following imaging parameters: 95 kV, 5.2 mA, 15 × 15 cm field of view, and 0.2 mm voxel size. Images were analyzed with the aid of NNT Viewer software (version 2.21; Image Works, Verona, Italy).

All images were independently evaluated by two calibrated examiners (Narges Simdar and Seyed Mohsen Helmi). Prior to the main assessment, both examiners were calibrated on 30 randomly selected CBCT images. Agreement between and within examiners was determined using the Cohen’s kappa statistic, revealing excellent agreement (interexaminer *κ* = 0.87–0.92 and intraexaminer *κ* = 0.89–0.94). In cases of disagreement, consensus was reached through discussion with the third author (Javad Mesri).

**Figure 1:**
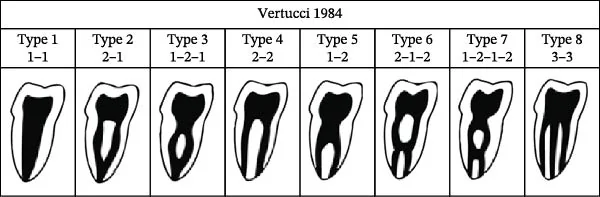
Root canal configurations of mandibular anterior teeth according to Vertucci’s classification [11].

Using NNT software, a line parallel to the long axis and a line perpendicular to it were drawn. Axial, sagittal, and coronal slices (0.20–0.25 mm thickness) were examined. Image brightness and contrast levels were modified in the software when necessary to improve visualization. Data recorded included tooth location (right or left), number of roots and canals, morphology (based on Vertucci’s classification, Figure 1), and symmetry of the root canal system. The study defined symmetry as the existence of the same Vertucci type and the same number of roots in the contralateral teeth of the same type.

Statistical analyses were performed using SPSS version 24 (SPSS Inc., Chicago, IL, USA). The normality of data distribution was first examined using the Kolmogorov–Smirnov test. Descriptive statistics were reported as the mean ± standard deviation for continuous variables and as frequency (percentage) for categorical variables. Comparisons between genders and between right and left sides were performed using the independent samples *t*‐test or Mann–Whitney *U* test for continuous data and the chi‐square test or Fisher’s exact test for categorical data, as appropriate. A significance level of 0.05 was set.

## 3. Results

In this study, data from 852 teeth in 148 patients were included in the analysis. The age of the participants was 36.76 ± 9.67 years; 44.6% (66) of participants were male and 55.4% (82) were female.

**Figure 2:**
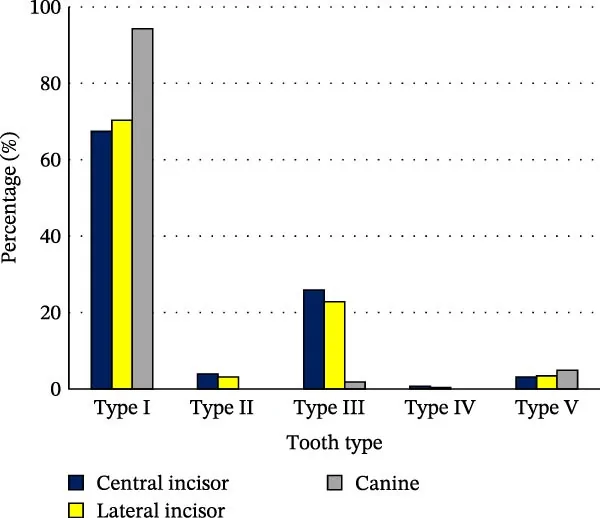
Distribution of root canal configurations according to Vertucci’s classification in mandibular central incisors, lateral incisors, and canines.

The frequency of canal morphology based on Vertucci’s classification [11] is presented in Table 1 and Figure 2. The most prevalent canal morphology was type I (67.4%), while type IV was the least common (0.7%) among the 282 central incisors examined. In the 290 lateral incisors, type I morphology was also the most frequent (70.3%), with type IV being the least frequent (0.4%). For the 280 canines assessed, type I was again the most common morphology (94.3%), whereas type III was the least frequent (1.8%).

**Table 1:** Frequency of canal morphology according to Vertucci classification.

Tooth type	Type I	Type II	Type III	Type IV	Type V
Central incisors	67.4% (190)	3.9% (11)	25.9% (73)	0.7% (2)	3.1% (6)
Lateral incisors	70.3% (204)	3.1% (9)	22.8% (66)	0.4% (1)	3.4% (10)
Canines	94.3% (264)	0% (0)	1.8% (5)	0% (0)	4.9% (11)

*Note:* No significant relationship was found between gender and canal morphology type in centrals, laterals, and canines (*p* = 0.588, *p* = 0.403, and *p* = 0.555, respectively). Additionally, no significant relationship was observed between tooth location and canal morphology in centrals, laterals, and canines (*p* = 0.964, *p* = 0.898, and *p* = 0.610, respectively).

**Figure 3:**
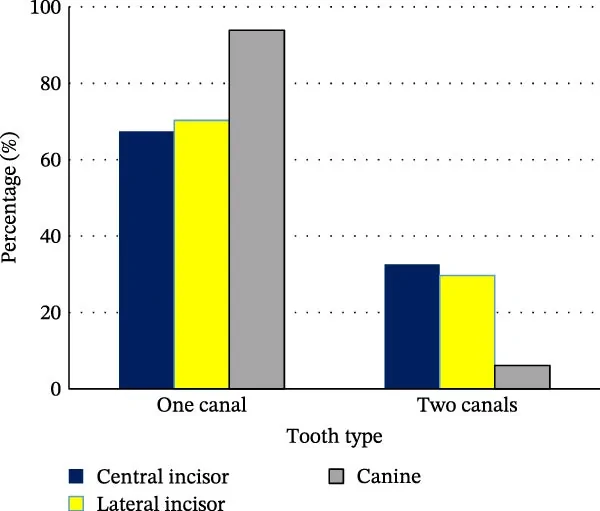
Prevalence of single root canal versus two root canals in mandibular anterior teeth.

Among the central incisors, 67.4% (190) had one root canal, while 32.6% (92) had two root canals. For lateral incisors, 70.3% (204) had one root canal, and 29.7% (86) had two root canals. In canines, 94.3% (264) had one root canal, and 6.1% (16) had a second canal. Figure 3 illustrates the prevalence of single versus two root canals. The frequency of centrals, laterals, and canines with a single canal was significantly higher than those with two canals (*p* < 0.001 for all comparisons).

No significant relationship was observed between gender and the number of canals in centrals, laterals, and canines (*p* = 0.911, *p* = 0.236, and *p* = 0.261, respectively). Likewise, no significant relationship was observed between tooth location and the number of root canals in these teeth (*p* = 1.000, *p* = 1.000, and *p* = 0.873, respectively).

All centrals and laterals had one root, while 96.8% (271) of canines had one root and 3.2% (9) possessed two roots. Most canines had a single root, and the proportion of single‐rooted canines was significantly higher than two‐rooted ones (*p* < 0.001). No significant relationship was observed between the number of roots in canines and gender (*p* = 0.429), nor between tooth location and the number of tooth roots in canines (*p* = 0.559).

The results indicated that 90.1% (254) of central incisors had symmetrical roots, while 9.9% (28) had asymmetric roots. In lateral incisors, 93.1% (270) had symmetrical roots and 6.9% (20) had asymmetric roots; in canines, 91.4% (256) had symmetrical roots and 8.6% (24) had asymmetric roots. No significant relationship was found between gender and root symmetry in centrals, laterals, and canines (*p* = 0.581, *p* = 0.337, and *p* = 0.549, respectively). Additionally, no significant relationship was found between tooth location and root symmetry in these teeth (*p* = 1.000 for centrals and laterals; *p* = 0.723 for canines).

## 4. Discussion

The present study indicates that the most prevalent type of canal morphology among all examined teeth was type I according to Vertucci’s classification. This finding aligns with recent multiethnic studies showing type I prevalence ranging from 65 to 75% across populations [18, 19] and is in agreement with studies conducted by Mashyakhy [20] and Sroczyk‐Jaszczyńska et al. [21] who reported similar results in Arabic and Polish populations, respectively. Geduk et al. [22] and Altunsoy et al. [19] also identified type I as the most prevalent canal morphology in a Turkish population. Furthermore, Verma et al. [18] and Kamtane and Ghodke [23] reported comparable findings in an Indian population. In a related study, Aminsobhani et al. [6] found that 72.3% of centrals, 70.6% of laterals, and 71.8% of canines in an Iranian population exhibited type I canal morphology according to Vertucci’s classification. No significant relationship was observed between canal morphology and gender in this study, which aligns with previous research [4, 10, 18, 22, 24–26].

The second most prevalent configuration in mandibular incisors was Vertucci Type III (25.9% in central incisors and 22.8% in lateral incisors), accounting for ~79%–80% of teeth with two root canals. This 1‐2‐1 pattern, where a single canal bifurcates in the mid‐root and merges again apically, is clinically significant. The lingual branch of the canal is often hard to identify and adequately treat using conventional techniques due to its narrow anatomy and superimposition on periapical radiographs, potentially leading to incomplete debridement, persistent infection, or endodontic failure [4, 5]. These findings further highlight the value of preoperative CBCT in identifying such configurations to improve the predictability and success of root canal treatment.

In this investigation, both incisors and canines predominantly exhibited a single root canal. Specifically, 32.6% of centrals, 29.7% of laterals, and 6.1% of canines were observed to possess a second root canal. These percentages closely match reports from neighboring regions, including Turkey [22] and Arab countries [20]. Herrero‐Hernández et al. conducted a systematic review on the occurrence of a secondary canal in the incisors of mandible and reported that the occurrence of a second canal may vary from 0.4% to 45% [4]. Similarly, Mashyakhy [20] found that 26.3% of centrals and 30.8% of laterals had a second canal in an Arabic population. Sroczyk‐Jaszczyńska et al. [21] reported that 33.7% of centrals, 32.5% of laterals, and 14% of canines had two root canals in a Polish population. Geduk et al. [22] also noted that 35.6% of incisors had two root canals. No significant relationship between gender and the number of canals was observed in this study, which corroborates findings from previous research [4, 6, 18, 22, 24].

In this study, all laterals and centrals, as well as 96.8% of canines, were found to have a single root. This finding is consistent with the results reported by Sroczyk‐Jaszczyńska et al. [21], who reported that all centrals and laterals in a Polish population had one root, with only 2.4% of canines exhibiting two roots. Verma et al. [18] reported that all incisors of mandible in an Indian population had a single root, while Kamtane and Ghodke [23] similarly reported that all incisors of mandible in an Indian population were single‐rooted. Geduk et al. [22] found that all mandibular incisors in a Turkish population had one root. Aminsobhani et al. [6] reported that 100% of mandibular incisors and 96.3% of canines in an Iranian population had one root, which supports our findings. In contrast to these studies, Zhengyan et al. [24] reported that 0.3% laterals and 0.8% of canines had two roots in a Chinese population. This discrepancy may be attributed to the differing ethnic backgrounds of the study populations. No significant relationship was identified between gender and the number of roots in this study, which aligns with previous research [6, 10, 25, 26].

The current study demonstrated that 90.1%, 93.1%, and 91.4% of centrals, laterals, and canines, respectively, exhibited symmetrical roots. This high bilateral symmetry is clinically relevant because when contralateral teeth show similar canal configurations, clinicians can better predict the anatomy of the opposite side and improve treatment planning. However, the ~7%–10% asymmetry rate indicates that clinicians should not assume complete similarity and must carefully evaluate each tooth independently. This high symmetry rate is consistent with the findings from anatomical studies in Asian populations [26, 27]. A study conducted by de Oliveira‐Santos et al. using micro‐CT technology confirmed that symmetry in anterior teeth of mandible is a common feature, with variations primarily attributed to developmental anomalies rather than ethnic differences [28]. Mashyakhy [20] reported that 91.2% of centrals and 85.8% of laterals displayed symmetry. Similarly, Lin et al. [27] found that 95.2% of centrals and 93.8% of laterals had symmetrical roots. Additionally, the study conducted by Kayaoglu et al. [26] demonstrated that 95.5% of mandibular canines had symmetrical roots. In recent years, CBCT has proven valuable not only for root canal studies but also for evaluating critical anatomical relationships in the mandible. For instance, El Hadidi et al. [29] used this imaging modality to analyze the position of the inferior alveolar nerve relative to mandibular posterior teeth, underscoring its reliability for preoperative planning. Similarly, finite element analysis studies have explored biomechanical aspects of distractors in managing facial asymmetry following ankylosis, highlighting the growing role of advanced imaging and modeling in complex mandibular cases [30].

The prevalence of single‐rooted mandibular incisors (100%) and canines (96.8%) in the present Iranian sample is consistent with most previous CBCT studies from Turkey [19], Poland [21], India [18], Saudi Arabia [20], and China [24], where single‐rooted incisors approached or reached 100% and canines exceeded 97%. Similarly, the rate of a secondary canal in centrals (32.6%) and laterals (29.7%) falls within the ranges reported in these populations (26%–36% for central and 28%–34% for lateral incisors). The high symmetry rates observed (90.1%–93.1%) are also comparable to findings in other ethnic groups, supporting the notion that mandibular anterior teeth exhibit relatively stable and symmetrical anatomy across diverse populations.

The present study is subject to certain limitations. It was designed as a retrospective single–center investigation using convenience sampling from patients referred to one university clinic, which may limit its generalizability to the entire Iranian population. Moreover, although high‐resolution CBCT was used, very fine anatomical details smaller than the voxel size might not have been detected. Finally, since the sample included only Iranian individuals, caution should be exercised when extrapolating these results to other ethnic groups.

In summary, this study found that all lower incisors had one root, predominantly exhibited a single root canal, and mostly displayed symmetrical roots. Based on Vertucci’s classification, type I was the predominant root canal morphology. Most canines exhibited a single root and canal, along with a high degree of symmetry.

## Author Contributions

**Narges Simdar**: conceptualization, methodology, investigation, resources, data curation, writing – original draft, software, visualization. **Javad Mesri (corresponding author)**: conceptualization, supervision, project administration, validation, writing – review and editing, formal analysis. **Seyed Mohsen Helmi**: investigation, formal analysis, data curation.

## Funding

This study did not receive any specific funding.

## Disclosure

All authors have read and approved the final version of the manuscript. The corresponding author had full access to all of the data in this study and takes complete responsibility for the integrity of the data and the accuracy of the data analysis.

## Ethics Statement

The study received approval from the Ethics Committee of Guilan University of Medical Sciences (IR.GUMS.REC.1399.358).

## Conflicts of Interest

The authors declare no conflicts of interest.

## Supporting Information

Additional supporting information can be found online in the Supporting Information section.

## Supporting information


**Supporting Information** Figure S1: Flow diagram of the study participants and CBCT scans selection process according to the inclusion and exclusion criteria.

## Data Availability

The data that support the findings of this study are available from the corresponding author upon reasonable request.
