# Supplementary material for: Evaluation of Symmetry and Morphology in Mandibular Permanent Anterior Teeth Using Cone‐Beam Computed Tomography in an Iranian Population
Source: Int J Dent. 2026 Aug 20;2026:9659582. doi: 10.1155/ijod/9659582 (PMC13494349; doi:10.1155/ijod/9659582)
Supplement: Supplementary file 1 [file IJOD-2026-9659582-s001.docx]

No

Yes

Patients Referred for CBCT

Imaging

2018–2021

CBCT Scans Screened

Eligibility Criteria Met?

Excluded

Incomplete root

formation, Previous RCT,

Periapical lesions, Root

resorption,

Deep caries, Calcified

canals

148 CBCT Scans Included

852 Mandibular Anterior

Teeth:

282 Central Incisors

290 Lateral Incisors

280 Canines

Final Analysis

Root and Canal Morphology

+

Symmetry Assessment

Supplementary Figure 1. Flow diagram of the study participants and CBCT scans selection process according to the inclusion and exclusion criteria
